# Diabetic Ketoacidosis as the First Manifestation of Ectopic Cushing’s Syndrome

**DOI:** 10.7759/cureus.98305

**Published:** 2025-12-02

**Authors:** Regina Medeiros, Vitor Carneiro, Isabel Sousa, Bernardo Dias Pereira

**Affiliations:** 1 Endocrinology and Nutrition, Hospital Divino Espírito Santo-EPER (Entidade Pública Empresarial Regional), Ponta Delgada, PRT; 2 Pathology, Hospital Divino Espírito Santo-EPER (Entidade Pública Empresarial Regional), Ponta Delgada, PRT

**Keywords:** cushing's syndrome, diabetic ketoacidosis, ectopic cushing’s syndrome, metyrapone, neuroendocrine tumor

## Abstract

Diabetic ketoacidosis is an exceptionally rare initial manifestation of ectopic adrenocorticotropic hormone (ACTH) syndrome. A 42-year-old woman with multiple cardiovascular risk factors was admitted to the emergency room with diabetic ketoacidosis. During stabilization, florid Cushing’s syndrome was suspected and confirmed biochemically as ACTH-dependent. Further biochemical and imaging surveys led to the diagnosis of a 25x15 mm nodule in the lingula. Thoracic surgery was performed, and pathology revealed a neuroendocrine tumor positive for ACTH.

We reviewed eight additional cases of diabetic ketoacidosis associated with Cushing’s syndrome from PubMed. Clinicians should bear in mind this etiology of diabetic ketoacidosis based on clinical signs and younger patients with multiple, age-atypical comorbidities. This would permit the expedited targeted stabilization of Cushing’s syndrome and the suitable institution of the diagnostic approach and treatment for this challenging syndrome.

## Introduction

Endogenous Cushing’s syndrome (CS) is a rare disease resulting from pathological glucocorticoid excess of neoplastic origin, with an annual incidence of two/three cases per 1.000.000 inhabitants [1]. The severity of CS varies widely from mild to severe and, if left untreated, can be fatal due to the increased risk of cardiovascular events and opportunistic infections. Endogenous CS is classified as adrenocorticotropic hormone (ACTH)-dependent (80%) and -independent (20%) forms. ACTH-dependent CS is further divided into Cushing’s disease (68%) when the pituitary is the source of excess ACTH, or ectopic ACTH syndrome (EAS; 12%) when the cause is a non-pituitary neoplasia of neuroendocrine origin. EAS has an annual incidence of one case per 1.250.000 inhabitants and is more frequent in men [1]. It can be secondary to an aggressive small-cell lung carcinoma (19%), but the majority of cases arise from indolent lesions such as bronchial and thymic (combined: 33%) or pancreatic (12%) neuroendocrine tumors (NET) [1-3]. These indolent lesions usually evolve clinically over 6 to 24 months, whereas carcinomas have a faster onset. Symptoms and signs of excess cortisol in EAS are usually indistinguishable from Cushing’s disease. The most discriminatory signs of CS are plethora, purplish striae, proximal myopathy, and spontaneous ecchymosis. Multiple vascular risk factors, namely, hypertension, diabetes mellitus (DM), dyslipidemia, and obesity (especially central adiposity), occurring in a young patient, should also raise suspicion for CS [2]. Diabetic ketoacidosis (DKA) as the inaugural presentation of CS is very rare [1-3]. We searched through PubMed and reviewed articles in English where this association was reported using keywords such as “Cushing's syndrome”, “Diabetic ketoacidosis”, “hypercortisolism”, and “Ectopic ACTH syndrome”. CS presenting initially with DKA is, as to this day, limited to eight case reports [4-11]. The clinical recognition of this syndrome as a very rare etiology of DKA is of paramount importance, as it is usually severe and relates to sepsis and several biochemical, hematologic, and hemodynamic derangements that should be addressed expeditiously with targeted drugs [3].

Here, we describe a female patient with florid clinical EAS uncovered upon her admission to the Emergency Room (ER) due to DKA. We searched through PubMed and reviewed articles in English where this association was reported, using keywords such as “Cushing's syndrome”, “Diabetic ketoacidosis”, “hypercortisolism”, and “Ectopic ACTH syndrome”.

This article was previously presented as a meeting abstract at the 2024 ENDO, The Endocrine Society Annual Meeting on June 3, 2024.

## Case presentation

**Figure 1:**
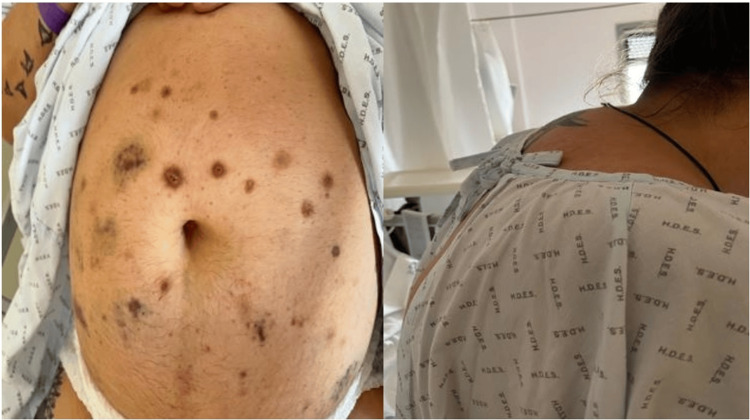
Patient's Cushingoid features

A 42-year-old woman was admitted in June 2022 to the ER due to severe DKA and hypokalemia (Table 1) and mild coronavirus disease. Physical examination at initial presentation was also remarkable for grade 2 hypertension with hypertensive retinopathy. Florid Cushingoid features, including a “buffalo hump”, plethora, hirsutism, abdominal ecchymosis, and marked proximal limb sarcopenia were noted (Figure 1).

The patient was transferred to the intensive care unit (ICU). A multimodal treatment plan was initiated, including intravenous insulin (total daily dose: 1.2U/Kg) as per the protocol for DKA, antihypertensives, and prophylactic doses of low-molecular-weight heparin. After resolution of DKA and hydroelectrolytic disturbances, a gasometric follow-up revealed metabolic alkalosis (pH 7.529). The patient was then able to report a six-month history of weight gain, secondary amenorrhea, impaired concentration and memory, ecchymoses, and proximal myopathy with frequent falls and dependency on relatives for daily life activities. No chronic diarrhea or flushing was reported. She also reported a fungal pneumonia, dyslipidemia, and hypertension in the last four months, and a diagnosis of DM treated with metformin two weeks before her admission to the ER. Family history was unremarkable. Biochemical surveys (Table 1) revealed ACTH-dependent hypercortisolism, low thyroid-stimulating hormone (TSH), and hypogonadotropic hypogonadism. High-dose dexamethasone suppression (HDDS) and corticotropin-releasing hormone (CRH) stimulation tests were not suggestive of a pituitary source of ACTH (Table 1). Pituitary magnetic resonance imaging was normal. While waiting for further investigations regarding the source of excess ACTH, the patient was started on 750 mg/day of metyrapone in three divided doses. The patient was started and discharged from the ward with hydrocortisone 10 mg in the morning and 5 mg at midday and in the afternoon. The dose of metyrapone was carefully adjusted during two months according to morning serum cortisol, but was rapidly decreased and stopped due to spontaneous clinical resolution of CS. In the postoperative follow-up (total: 23 months), Cushingoid features (plethora, dorsal fat pad, ecchymosis, central adiposity) continued to disappear, and she regained muscle mass and independence in her daily activities and remission from all glucocorticoid related-comorbidities was maintained (fasting glucose: 91 mg/dL; glycated hemoglobin (HbA1c): 5.8%; low-density lipoprotein (LDL) cholesterol: 138 mg/dL; triglycerides: 80 mg/dL). Twelve months after surgery, the patient was able to discontinue hydrocortisone upon biochemical evidence of restoration of adrenal function (cortisol peak at Synacthen test: 21.1 ug/dL; basal ACTH: 15.6 pg/mL). Her last (23 months after surgery) endocrine surveys (midnight salivary cortisol: 0.14 ug/dL; ACTH: 18 pg/mL) and thoracic CT showed no evidence of disease relapse.

**Table 1:** Biochemical surveys of the patient at baseline and at the 12-month follow-up * After metyrapone washout ¥ Gonadotropins not repeated due to resumption of regular menses Abbreviations: ACTH, adrenocorticotropic hormone; CRH, corticotropin-releasing hormone; DST, dexamethasone suppression test; FSH, follicle-stimulating hormone; HbA1c, hemoglobin A1c; HDDS, high-dose dexamethasone suppression; IGF-1, insulin-like growth factor type 1; LH, luteinizing hormone; TSH, thyroid-stimulating hormone; UFC, urinary free cortisol

Parameter	Presentation	12-month follow-up	Reference
Hemoglobin (g/dL)	12.8	-	12-15.5
White blood count (×103/uL)	11.3	-	4.0-11.5
Platelets (×103/uL)	331	-	150-400
Fasting blood glucose (mg/dL)	427	76	74-106
HbA1c (%)	9.6	5.6	<6.5
Serum sodium (mmol/L)	146	-	135-145
Serum potassium (mmol/L)	2.7	-	3.5-5.1
Serum creatinine (mg/dL)	0.32	0.59	0.67-1.17
pH	7.17	-	7.35-7.45
HCO3^- ^(mmol/L)	4.4	-	21-26
Anion gap	35	-	7
IGF-1 (ng/mL)	89.8	-	77-234
FSH (mUI/mL)	0.9	-^¥^	3.5-12.5
LH (mUI/mL)	<0.1	-^¥^	2.4-12.6
Prolactin (ng/mL)	8.8	-	4.0-24.3
TSH (UI/mL)	0.02	0.61	0.35-4.94
Free T4 (ng/dL)	1.26	1.02	0.7-1.48
Midnight salivary cortisol (ug/dL)	25.5	2.4*	<7.5
UFC (ug/dL)	1072.5	74.5*	<176
Cortisol at 1 mg overnight DST (ug/dL)	25.7	-	<1.8
Cortisol, baseline (ug/dL)	30.9	11.4*	5-18
Cortisol after HDDS test (ug/dL)	42.1	-	Refer to reference 2
ACTH, baseline (pg/mL)	93.4	22.1*	7.2- 63.3
ACTH, maximum after CRH (pg/mL)	101.8	-	Refer to reference 2

**Figure 2:**
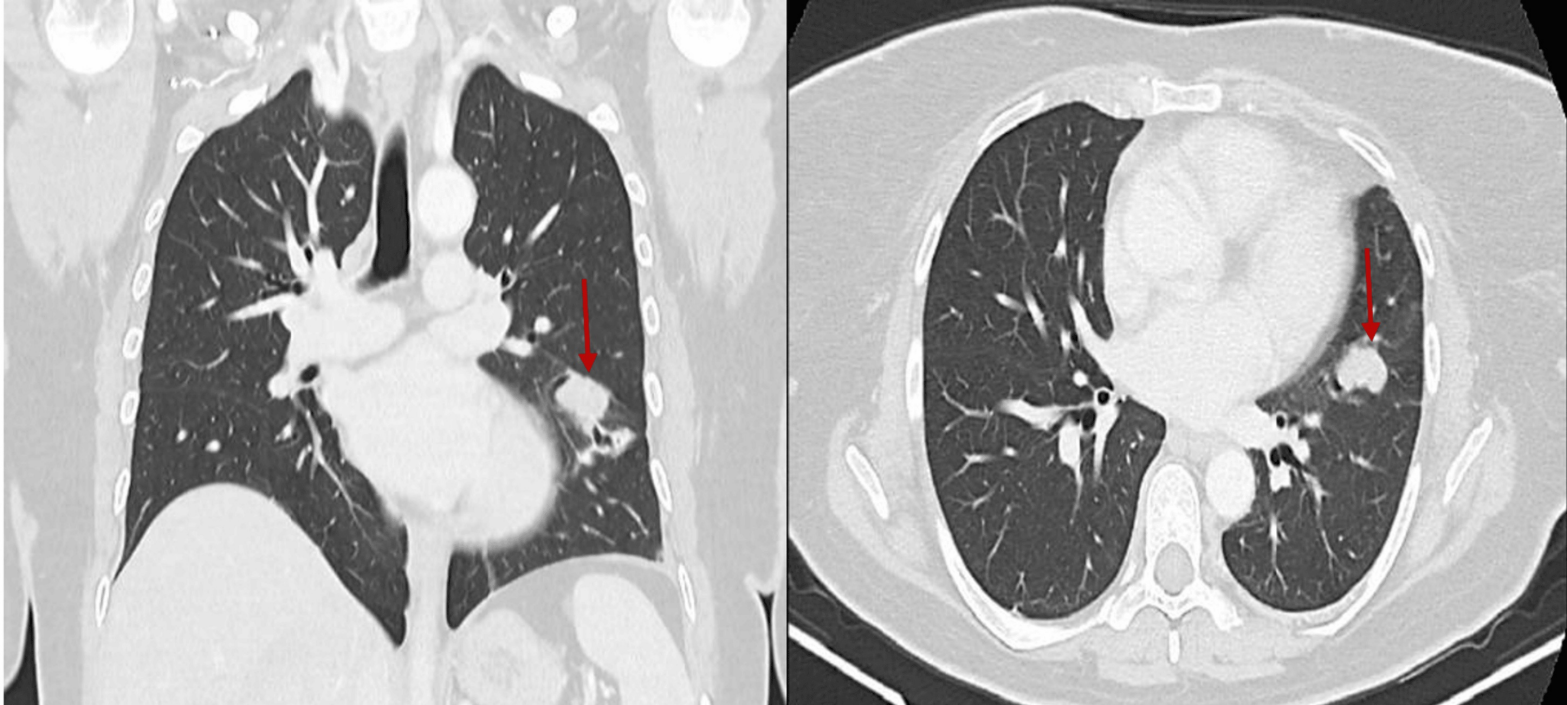
Thoracic CT scan revealed a 25x15 mm, well-defined nodule in the lingula

She was referred for inferior petrosal sinus sampling (IPSS) but it was postponed for several months due to healthcare strikes. While waiting for IPSS, she performed a thoracic computerized tomography (CT) scan to exclude EAS, which revealed thymic hyperplasia and a 25x15 mm, well-defined nodule in the lingula (Figure 2).

**Figure 3:**
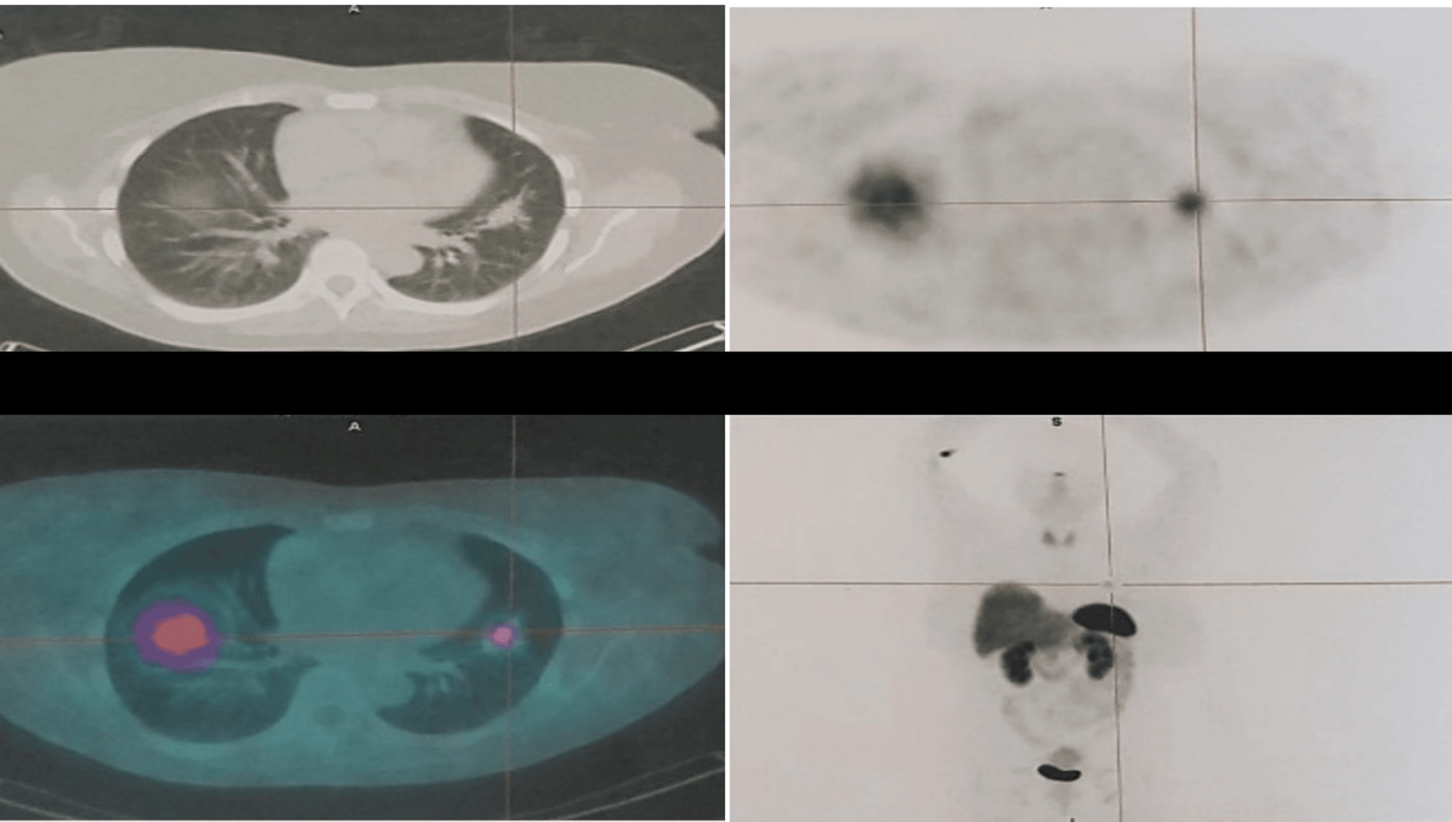
68Ga-DOTANOC PET/CT showing a single uptake in the lingula. Abbreviations: PET/CT, positron emission tomography-computed tomography

A ^68^Ga-DOTANOC positron emission tomography-computed tomography (PET/CT) was then performed and showed a single uptake in the same lung region (Figure 3).

**Figure 4:**
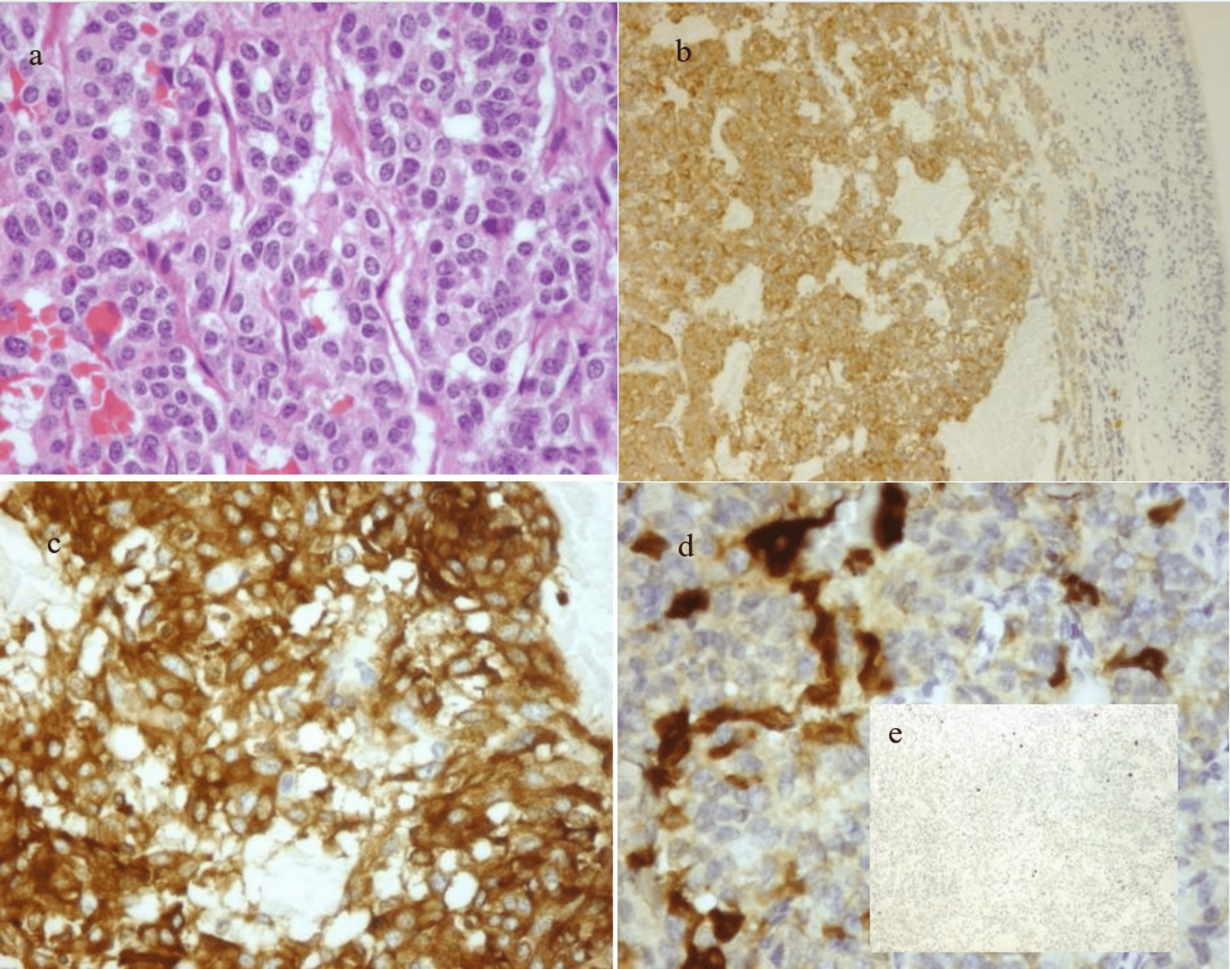
Immunohistochemistry findings a- hematoxylin and eosin x400 magnification, b- synaptophysin x100 magnification, c- chromogranin A x400 magnification, d- ACTH x400 magnification, e- Ki-67 x100 magnification.

The patient was referred to thoracic surgery and underwent lingulectomy plus excisional biopsy of the interlobar lymph nodes. Pathology revealed a typical carcinoid/neuroendocrine tumor (NET), grade one (Ki67<2% and <2 mitosis per high-power field (HPF)) without involved lymph nodes, which showed positivity for ACTH (Figure 4).

The patient was started on hydrocortisone 10 mg in the morning and 5 mg at midday and afternoon, which was discontinued 11 months later due to restoration of adrenal function (cortisol peak at Synacthen test: 21.1 ug/dL; basal ACTH: 15.6 pg/mL). In the postoperative follow-up, Cushingoid features continued to disappear, and she regained muscle mass and independence in her daily activities. Her last CT showed no evidence of disease.

## Discussion

Severe CS (SCS) is defined by random serum cortisol above 41 ng/dL and/or a urinary free cortisol (UFC) more than fourfold the upper limit of normal and/or severe hypokalemia (<3.0 mmol/L), along with the recent onset of one or more of the following: sepsis, opportunistic infection, refractory hypokalemia, uncontrolled hypertension, edema, heart failure, gastrointestinal bleeding, glucocorticoid-induced acute psychosis, progressive debilitating myopathy, thromboembolism, uncontrolled hyperglycemia and ketoacidosis [3]. SCS results in high morbidity and mortality, requiring a rapid recognition and targeted therapy of the uncontrolled hypercortisolism [3]. Patients with SCS usually have florid signs, and straightforward clinical suspicion is possible, except in cases of ECS due to small-cell lung carcinoma, where the rapid onset of hypercortisolism and related morbidity precedes the development of clinical stigmata [2,3]. The gasometric parameters in DKA associated with SCS can also provide clues for the presence of CS. The mineralocorticoid effect of excess cortisol leads to metabolic alkalosis through increased hydrogen excretion in the distal nephron, which is masked by metabolic acidosis due to excess β-hydroxybutyrate and acetoacetate [6,12,13]. This mixed acid-basic disorder can be suspected by a ratio of ∆anion gap to ∆HCO3 of higher than one, which is not seen in pure metabolic acidosis. Additionally, after treating the DKA by decreasing ketones through the inhibition of its production by insulin and increased renal excretion with improved renal perfusion, metabolic alkalosis may supervene in gasometric monitoring, as seen in our report and others [6,9]. In rare cases, SCS can also lead to diabetic ketoalkalosis instead of DKA [1]. Several factors may contribute to the predominant alkalosis, namely, decreased hydrogen due to high renal excretion (excess mineralocorticoid effect), intracellular shift (due to severe hypokalemia), gastrointestinal losses (vomiting), and hyperventilation due to pulmonary diseases (as in heavy smokers) [13,14].

The main priorities in managing SCS are to control opportunistic infections, hypokalemia, DM, hypertension, and psychosis, and, importantly, investigations of the etiology of CS should be postponed until clinical stabilization [3]. The control of glucocorticoid-induced complications should encompass therapies to stabilize/reverse the CS induced morbidity (e.g., large-spectrum antibiotics for opportunistic infections; spironolactone for hypokalemia; insulin for DM) followed by targeted treatment of hypercortisolism [3]. Several oral adrenolytic agents are available and have proved their usefulness in SCS, namely, metyrapone (onset: hours; UFC normalization: 83%), ketoconazole/levoketoconazole (onset: days; UFC normalization: 70-81%), osilodrostat (onset: hours; UFC normalization: 82%), and mitotane (onset: days to weeks; UFC normalization: 72-82%). They can be used in monotherapy or in combination therapy, the latter strategy increasing the efficacy with lower doses of drugs and a lower risk of side effects [3,14]. Additionally, as first-line therapy for patients with an unavailable oral route (e.g., glucocorticoid-induced psychosis), or as second-line therapy when other adrenolytic agents have failed to control hypercortisolism, the anesthetic etomidate can be used under multidisciplinary supervision in an ICU, and it is highly effective (~100%) in controlling SCS within hours, in doses that do not induce anesthesia [3]. If medical therapy proves unsuccessful, bilateral adrenalectomy may be considered after careful clinical judgement, as it is highly effective in life-threatening SCS uncontrolled by medical therapy. Nevertheless, all attempts should be made to reduce hypercortisolemia with medical therapy before surgery [3].

DKA, as the inaugural presentation of CS, was previously published in eight case reports [4-11] (Table 2). Briefly, and including our case, almost all reports were severe (77.8%), mainly from EAS (55.6%) or pituitary adenomas (33.3%), and with a female preponderance (77.8%).

**Table 2:** Review of published cases of DKA as the inaugural presentation of CS *Deceased Abbreviations: ACTH, adrenocorticotropic hormone; CS, Cushing’s syndrome; EAS, ectopic ACTH syndrome; NET, neuroendocrine tumor

Reference	Gender	Age	Florid CS signs	Severe CS	Etiology of CS	Definitive treatment
Uecker JM, et al. [4]	Female	43	Yes	Yes	EAS (duodenal NET)	Pancreaticoduodenectomy
Kahara T, et al. [5]	Male	53	No	No	ACTH-independent	Adrenalectomy
Weng Y, et al. [6]	Female	28	Yes	Yes	Cushing’s disease (macroadenoma)	Transsphenoidal surgery
Catli G, et al. [7]	Female	16	Yes	Yes	Cushing’s disease (microadenoma)	Transsphenoidal surgery
Sakuma I, ​​​​​​et al. [8]	Female	56	Yes	Yes	EAS (pheochromocytoma)	Adrenalectomy
Achary R, et al. [9]	Female	48	Yes	Yes	Cushing’s disease (microadenoma)	Transsphenoidal surgery
Cheong H, et al. [10]*	Female	22	Unknown	Unknown	EAS (medullary thyroid carcinoma)	None
Shangjian L, et al. [11]	Male	46	Unknown	Yes	EAS (pheochromocytoma)	Adrenalectomy
Our case	Female	42	Yes	Yes	EAS (bronchial NET)	Thoracic surgery

The etiology of CS should be investigated in diagnostic steps. After confirming hypercortisolism (low-dose dexamethasone suppression test, UFC, and/or late-night salivary cortisol) and its ACTH dependence (usually well above 20 pg/mL in EAS), the source of excess ACTH should be pursued. The CRH test is the most accurate dynamic test to distinguish between pituitary and ectopic sources of ACTH, followed by the desmopressin and HDDS tests. The combination of CRH and HDDS tests has an accuracy close to the IPSS, the gold standard to distinguish pituitary from ectopic sources of ACTH. If the investigation approach points to EAS, the most accurate exam to detect a lesion is ^68^Ga-DOTA-somatostatin analogue PET/CT, followed by ^18^F-FDG PET and conventional cross-sectional imaging [1-3].

After being discharged from the ward, our patient showed spontaneous resolution of hypercortisolism requiring the withdrawal of metyrapone and all medications to control glucocorticoid-induced morbidity, suggesting cyclic CS. This very rare variant of CS is present when periods of hypercortisolism alternate with periods of normal cortisol secretion, each phase lasting from days to years, which makes this type of CS very challenging to manage. The pituitary is the main source of cyclic CS, followed by EAS and, infrequently, the adrenal gland. The criteria of three peaks and two periods of normal or low cortisol levels needed to diagnose cyclic CS were not seen in the follow-up period of our patient, as after one peak and trough, we found and removed the source of EAS [1].

## Conclusions

In the context of DKA, florid Cushing signs and multiple vascular risk factors occurring in a young patient should raise suspicion for Cushing’s Syndrome. The severity of this syndrome varies widely from mild to severe and, if left untreated, can be fatal due to the increased risk of cardiovascular events and opportunistic infections. Diabetic ketoacidosis precipitated by an endogenous excess of glucocorticoid is usually associated with severe Cushing’s syndrome and more frequently with EAS, which can have an abrupt onset. Prompt recognition and targeted stabilization of severe Cushing’s syndrome are crucial and should precede a definitive etiologic investigation.
